# Analysis of Related Factors of Brittle Hip Fracture in Postmenopausal Women with Osteoporosis

**DOI:** 10.1111/os.12605

**Published:** 2020-01-13

**Authors:** Hua‐feng Zhuang, Pei‐wen Wang, Yi‐zhong Li, Jin‐kuang Lin, Xue‐dong Yao, Hao Xu

**Affiliations:** ^1^ Department of Orthopaedics The Second Affiliated Hospital of Fujian Medical University Quanzhou China

**Keywords:** 25‐hydroxyvitamin D, Bone mineral density, Hip fracture, Osteoporosis

## Abstract

**Objective:**

To investigate the effects of age, body mass index (BMI), bone mineral density (BMD), and levels of serum 25‐hydroxyvitamin D (25OHD) on hip fracture on the condition of the bone density of femoral neck having reached the threshold of osteoporosis.

**Methods:**

A total of 252 postmenopausal women patients, whose bone density had reached the threshold of osteoporosis and age ≥50 years (50–98 years), collected from the Second Affiliated Hospital of Fujian Medical University from January 2015 to December 2018, were performed by retrospective analysis. According to whether or not they had a hip fracture, including femoral neck fracture or intertrochanteric fracture, the patients were divided into two groups, including 117 cases (50–84 years old) in the non‐hip fracture group and 135 cases (57–98 years old) in the hip fracture group. BMD was measured by Hologic Discovery A DXA bone mineral densitometer. Levels of serum 25OHD were detected by ROCHE detection instrument. Comparisons of age, BMI, bone density of femoral neck, and levels of serum 25OHD between the two groups were performed by using the Student's *t*‐test. Furthermore, the statistically significant factors were analyzed by multiple regression analysis to investigate the independent risk factors of hip fracture.

**Results:**

The group without hip fracture: 117 cases; average age: 67.4 ± 8.5 years; BMI: 22.3 ± 3.2 kg/m^2^; bone density of femoral neck: (0.504 ± 0.067) g/cm^2^; T‐value of femoral neck: −3.1 ± 0.6; levels of serum 25OHD: (24.9 ± 8.5) ng/mL. The group with brittle hip fracture: 135 cases; average age: 80.7 ± 7.6 years; BMI: 20.3 ± 3.5 kg/m^2^; bone density of femoral neck: (0.426 ± 0.077) g/cm^2^; T‐value of femoral neck: −3.8 ± 0.7; levels of serum 25OHD: (15.9 ± 8.9) ng/mL. Age, BMI, bone density of femoral neck, and 25OHD level of the group without hip fracture were markedly lower than hip fracture group (*P* < 0.05). The results of logistic regression analysis suggested that age, bone density of femoral neck, and levels of serum 25OHD were independent risk factors for fragile hip fracture on the condition of the bone density of femoral neck having reached the threshold of osteoporosis.

**Conclusion:**

Higher age, lower levels of bone density and 25OHD are the main risk factors of hip fracture on the condition of the bone density of femoral neck having reached the threshold of osteoporosis.

## Introduction

As a common complication of osteoporosis, hip fractures will be expected to affect up to 6.3 mn individuals worldwide in 2050, including 3.25 mn in Asia. Brittle hip fracture is a serious complication of osteoporosis. Leung *et al*. reported that 30% of patients died from various complications of osteoporosis hip fracture, and the disability rate is as high as 50% within 1 year after brittle hip fracture^1^. A report describes that the risk of death in the elderly can be sustained for 10 years after brittle hip fracture^2^. There are about 1 mn patients experiencing hip fracture in China every year, most of whom are elderly patients, and the incidence rate is rising rapidly. From 2002 to 2006, the incidence rate of hip fracture in China increased by 10% every year^3^. As age increases, the prevalence rate of hip fracture increases in over‐50‐year‐old people who display high mortality rates^4^, ^5^. Brittle hip fracture is closely related to osteoporosis. Bone mineral density (BMD) is not only the gold standard in the diagnosis of osteoporosis, but also an important index to evaluate fracture risk. Many studies have confirmed that low BMD and structural change of hip are vital causes of brittle hip fracture^6^, ^7^. The risk of hip fracture increases by 2.5 fold for every 1 standard deviation (SD) decrease in BMD of femoral neck. However, many fragile fractures occur in postmenopausal women with BMD over the threshold of osteoporosis. Previous studies have shown that there is no significant difference in BMD between patients aged 65–76 years who have reached the threshold of osteoporosis with and without fractures^8^. Clinically, it can also be seen that many patients whose femoral neck BMD has reached the threshold of osteoporosis do not suffer from hip fracture, and less than 50% of patients with hip fracture have the femoral neck BMD or total hip BMD that reaches the threshold of osteoporosis^7^, ^9^. Therefore, BMD is not the sole risk factor for hip fractures.

The hip fracture risk was increased by 40% for each SD decrease in serum 25(OH)D level. Several possible mechanisms have been proposed for the associations between serum 25(OH)D level and the risk of fractures. Low serum 25(OH)D level usually indicates vitamin D deficiency. First, vitamin D increases the serum calcium concentrations and stimulates osteoblasts to produce RANKL, which is a protein that stimulates osteoclastogenesis. Low serum 25(OH)D level may increase parathyroid hormone (PTH) level, which may result in bone loss. Calcium mobilization from the bone is regulated by both vitamin D and PTH. Second, vitamin D deficiency has also been shown to be related to low muscle mass and muscle weakness. Koeckhoven *et al*. used the data of the Amsterdam osteoarthritis cohort and found that serum 25OHD level was significantly associated with muscle strength^10^. Moreover, another study performed by Orces^11^ showed similar results: compared with subjects with normal muscle strength, the prevalence rates of 25OHD deficiency were 31% and 43% higher among men and women with muscle weakness. Third, many studies suggested an association between vitamin D insufficiency and falls. Snijder *et al*. reported that low serum 25OHD level was significantly associated with increased falls in elderly people^12^. Rothenbacher *et al*.^13^ performed a prospective population‐based cohort study and showed an association between serum 25OHD level and the risk of first fall when serum 25OHD level < 20 ng/mL. Fourth, there are numerous studies showing the association between serum 25OHD level and BMD. The cohort study performed by Swanson *et al*.^14^ showed that higher levels of serum 25OHD were associated with higher baseline BMD and slower bone loss at the hip. Steingrimsdottir *et al*. performed a prospective study of 5764 men and women, and showed that, compared with reference values (50–75 nmol/L), values <30 nmol/L were associated with significantly lower BMD of the femoral neck^15^.

Although many clinical factors are associated with brittle hip fracture, few studies have been conducted on fracture risk factors after BMD reaches the threshold of osteoporosis. It is of great clinical significance to identify these risk factors to prevent brittle hip fracture. Therefore, we aim to investigate several unanswered questions about the occurrence of brittle hip fracture after BMD reaches the threshold of osteoporosis. First, does BMD continue to affect the occurrence of brittle hip fracture? Second, are there any other factors besides BMD involved in brittle hip fracture? Third, what are the most significant risk factors among these independent factors analyzed by multivariate regression analysis? This study may provide guidance for effective clinical intervention.

Considering that brittle hip fractures are more likely to occur in older age, BMD is an important factor in predicting fractures. Ninety per cent of hip fractures are caused by falls, and vitamin D status is associated with falls. Therefore, age, BMI, femoral neck BMD, and serum 25OHD were used as observation indexes in this study.

## Patients and Methods

### *Patient Demographics*

Each patient provided informed consent for participation in the study. This retrospective study was conducted in accordance with the Declaration of Helsinki (Ethical Principles for Medical Research Involving Human Subjects) and was approved by the Ethics Committee of Second Affiliated Hospital of Fujian Medical University.

A total of 252 postmenopausal women patients with osteoporosis, collected from the inpatient department of Second Affiliated Hospital of Fujian Medical University from January 2015 to December 2018, were performed by retrospective analysis. According to whether or not they had a hip fracture, including femoral neck fracture or intertrochanteric fracture, they were divided into hip brittle fracture group and non‐hip fracture group. In the brittle hip fracture group, 135 cases were fresh fractures caused by falls; in the group without hip fractures, there were 117 cases.

#### *Inclusion and Exclusion Criteria*

The inclusion criteria for all patients were the following: (i) BMD of femoral neck T ≤ −2.5 SD; (ii) age ≥50 years; (iii) menopause ≥1 year; and (iv) the hip fracture is a fresh fracture that occurred within the previous 2 weeks. Exclusion criteria: (i) patients who had been treated with anti‐osteoporosis drugs and vitamin D supplementation; (ii) secondary osteoporosis patients; and (iii) patients with abnormal function of liver and kidney and other important organs.

### *Clinical Assessment*

Three indicators were measured for each patient enrolled in this study.

#### *Body Mass Index (BMI)*

BMI is the number obtained by dividing a person's weight in kilograms by their height in meters squared. It is an international standard that is commonly used to measure body fatness and health. BMI is a neutral and reliable indicator.

#### *Bone Mineral Density*

Bone mineral density (BMD) was measured by Hologic Discovery A DXA bone mineral densitometer in the standard position with machine accuracy <1% and 0.25% coefficient of variation (% CV). Considering that the subjects were hip fractures, BMD of the hip and lumbar spine was measured and bone mineral density of the femoral neck was taken as an index. According to World Health Organization (WHO) diagnostic criteria, BMD of the femoral neck T ≤ −2.5 SD was defined as osteoporosis.

#### *Serum 25‐hydroxyvitamin D Detection*

Serum 25‐hydroxyvitamin D (25OHD) is currently recognized as the best indicator of vitamin D status *in vivo*. In the morning, 3 mL fasting venous blood was extracted, and ROCHE detection instrument (instrument model: Cobas e 601) was used. Vitamin D Total reagent was produced by ROCHE Company. The detection method was electrochemiluminescence. Criteria for judging vitamin D levels: lack, <20 ng/mL; insufficiency, ≥20 ng/mL and <30 ng/mL; adequacy, ≥30 ng/mL.

### *Statistical Analysis*

All statistical analyses were performed using SPSS 18.0 software (IBM Software, Chicago, IL, USA). For quantitative variables, the data were expressed as mean ± SD, and compared by using the Student's *t*‐test. The risk factors of hip fracture were analyzed by unconditional logistic regression model. A value of *P* < 0.05 indicated a statistically significant difference.

## Results

### *Influence Factors of Hip Fracture*

In the group without hip fracture, 117 cases were aged (67.4 ± 8.5) years; BMI: (22.3 ± 3.2) kg/m^2^; BMD of femoral neck: (0.504 ± 0.067) g/cm^2^; T‐value: −3.1 ± 0.57, 25OHD: (24.9 ± 8.5) ng/mL, 86 cases (73.5%) had 25OHD > 24 ng/mL. Hip fracture group: 135 cases were aged (80.7 ± 7.6) years, significantly higher than non‐hip fracture group; BMI: (20.3 ± 3.5) kg/m^2^, lower than that of non‐hip‐fracture group; BMD of femoral neck: (0.426 ± 0.077) g/cm^2^; T‐value: −3.8 ± 0.68, which was significantly lower than that of non‐hip‐fracture group; 25OHD: (15.9 ± 8.9) ng/mL, which was significantly lower than that of non‐hip‐fracture group, of which 114 cases (84.4%) had 25OHD ≤ 24 ng/mL (Table 1).

**Table 1:** Comparison of age, BMI, BMD of femoral neck and 25OHD levels between non‐fracture and fracture groups (mean ± SD)

Indexes	Non‐fracture group (117 cases)	Fracture group (135 cases)	*P* value	t‐value
Age (years)	67.4 ± 8.5	80.7 ± 7.6	0.000	13.222
BMI (kg/m^2^)	22.3 ± 3.2	20.3 ± 3.5	0.000	4.702
25OHD (ng/mL)	24.9 ± 8.5	15.9 ± 8.9	0.000	8.156
BMD of femoral neck (g/cm^2^)	0.504 ± 0.067	0.426 ± 0.077	0.000	8.564
Femoral neck T score	−3.1 ± 0.6	−3.8 ± 0.7	0.000	8.803

### *Logistic Regression Analysis of Influencing Factors of Hip Fracture*

Age, serum 25OHD levels, and BMD are all independent influence factors for the occurrence of fragile hip fracture. Age, lower serum 25OHD levels and BMD will increase risk of hip fracture. In addition, BMI is not an independent influence factor for hip fracture (Table 2).

**Table 2:** Multivariate logistic regression analysis of pneumonia in fragile hip fractures

Value		*OR* (95%*CI*)	*P* value
Age	50–65 years	Reference	
	66–80 years	6.893 (2.181, 21.781)	0.001
	>80 years	72.459 (18.742, 280.129)	0.000
25(OH)D	≥30 ng/mL	Reference	
	20–30 ng/mL		0.931
	<20 ng/mL	4.457 (1.581, 12.566)	0.005
T score	−2.5 to −3.5	Reference	
	−4.5 to −3.5	2.921 (1.373, 6.217)	0.005
	≤−4.5	5.241 (1.316, 20.870)	0.019

## Discussion

In this study, we found that hip fracture patients were older, had lower BMD and 25OHD (*P* = 0.000) on the condition of the femoral neck BMD reaching the threshold of osteoporosis. Furthermore, these factors were independent risk factors for hip fragile fracture.

### *Relationships between Age or BMD and Fragile Hip Fracture*

Fragile hip fractures are predominant in elderly women. It has been reported that 75% of hip fracture patients are over 65 years of age, and those aged between 70–89 years are the high‐risk age group for fragile hip fractures^5^. From the age of 50 to 80 years, the risk of hip fracture increased by 30 times; BMD of femoral neck is an important factor in predicting the risk of hip fracture. As every standard deviation of BMD of the femoral neck decreases, the risk of hip fracture will increase by 2.6 times. Age is a strong dependent factor for the relationship between BMD and the risk of hip fracture. Even if the same BMD of femoral neck reaches the threshold of osteoporosis T ≤ −2.5 SD, diverse ages will make a 1.4% to 14.2% difference of the risk of hip fracture in 10 years^6^. BMD decreases rapidly in the early postmenopausal period. Bone loss mainly comes from cancellous bone. However, BMD decreases slowly after 65 years of age, and bone loss is mainly from cortical bone, which plays a major role in bone strength. The thinning of cortical bone will lead to a significant reduction of bone strength, which is an important factor for hip fracture in elderly people when they fall^16^, ^17^, ^18^.

This study confirms that the hip fracture group is older and the femoral neck BMD is lower on the condition of BMD reaching the osteoporosis threshold. The femoral neck BMD comprehensively reflects the density of cancellous bone and cortical bone of the femoral neck, which constitutes about 80% of the bone strength. Therefore, the reduction of BMD will inevitably lead to the reduction of bone strength. This study confirms that age and further reduction of BMD are still risk factors for fragile hip fracture even when the femoral neck BMD reaches the threshold of osteoporosis.

### *Relationship between Serum 25OHD Level and Brittle Hip Fracture*

Falls and tumbles are the strongest risk factor for fractures in elderly patients, with 90% of hip fractures having a history of such activity. Low level of serum 25OHD is prone to fall and fracture. Annweiler and Beauchet found that the number of elderly patients who experienced falls and tumbles with a serum 25OHD below 20 ng/mL significantly increased the risk of fracture^19^. Feng *et al*. reported that 25OHD was obviously associated with hip fractures in the elderly patients^20^. For each reduction in standard deviation, major osteoporotic fractures increased by 40%. Lv *et al*. found that 25OHD < 24 ng/mL markedly increased the risk of hip fracture in elderly patients^21^. This study found that the levels of 25OHD is less than 24 ng/mL in 84.4% of hip fracture patients, whereas the levels of 25OHD is greater than 24 ng/mL in 73.5% of non‐hip fracture patients, which supports the opinion on reduced serum 25OHD increasing the risk of hip fracture. Järvinen *et al*. proposed that lateral tumbles increase the risk of hip fracture by five times, and that if the patient hits the greater trochanter in their fall, the risk of hip fracture will increase by 30 times^22^. Focus on prevention of fracture should be shifted from the treatment of osteoporosis to the prevention of tumbles^22^. Many reports have confirmed that vitamin D supplementation can effectively prevent tumbles and reduce the risk of hip fracture^23^, ^24^.

### *Limitations*

This retrospective study has several limitations. First, the number of cases in this study is relatively small and cannot represent all people with BMD reaching osteoporosis. Secondly, the collection and recording of all patients' medical history is not carried out by the same person, and there may be omission or deviation in the medical history, resulting in a deviation of results.

### *Conclusion*

Age, BMD, and serum 25OHD level are independent factors that affect brittle hip fracture in postmenopausal women with osteoporosis. As age is an uncontrollable factor, to prevent osteoporotic hip fracture we should pay attention to the use of anti‐osteoporotic drugs such as bisphosphonate, which can effectively improve BMD and reduce fracture. Meanwhile, attention should also be paid to vitamin D supplementation, which can effectively improve serum 25OHD level, so as to reduce the incidence of hip in postmenopausal women with osteoporosis.
